# DNMT3A mutations and clinical features in Chinese patients with acute myeloid leukemia

**DOI:** 10.1186/1475-2867-13-1

**Published:** 2013-01-11

**Authors:** Quanyi Lu, Yamei Chen, Hang Wang, Zhipeng Li

**Affiliations:** 1 Department of Haematology, Zhongshan Hospital of Xiamen University, Xiamen, Fujian, 361004, China; 2 Department of Biomedical Sciences and the Key Laboratory of the Ministry of Education for Cell Biology and Tumor Cell Engineering, School of Life Sciences, Xiamen University, Xiamen, Fujian, China

**Keywords:** DNMT3A, Acute myeloid leukemia, Gene mutations

## Abstract

Mutations in DNA methyltransferase 3A (DNMT3A) gene were recently demonstrated in acute myeloid leukemia(AML). Approximately 20% patients with AML carry DNMT3A gene mutations and was associated with a poor clinical outcome. but its clinical implications in Chinese AML patients are largely unknown. We analyzed 101 adult AML patients in china and found 14 patients (13.9%) harboring this mutation. 9 patient with M5, 2 patients with M1, 2patient with M2 and 1 patient with M3. We identified 11 missense mutation,2 nonsense and 30 bp deletion encompassing *DNMT3A*. The most common of them was predicted to affect 882Arg(in 4 patients). Double mutations were detected in two cases.10 of 33(43.5%). *DNMT3A* mutations occurred more frequently in older (age > 50y,p < 0.05) and the outcome is too badly for these patients. *We concluded that DNMT3A* mutations are highly recurrent in AML and is associated with distinct clinical and biologic characteristics and seems to be a useful as a prognostic marker.

## Background

Acute Myeloid Leukemia (AML) is a heterogeneous malignancy that can be classified by morphological, cytogenetic and molecular genetic criteria, Currently, cytogenetic analysis at diagnosis allows for risk-stratification of AML into a useful prognostic marker and intermediate risk [1], To some extent, Treatment protocols are adapted in an attempt to improve survival and decrease treatment-related toxicity. Unfortunately, prognostic implications have not been reliably established for AML in the intermediate risk category. In recent years, molecular analysis has identified novel markers with prognostic relevance in this diverse group. For example, AML with internal tandem duplication (ITD) in the fms-like tyrosine- kinase-3 gene (FLT3) carries a poor prognosis [2,3]. Molecular studies have pinpointed recurrent somatic mutations in NPM1, CEBPα, TET2,MLL-PTD and fusion proteins such as PML-RARα and CBFB-MYH11, which characterize specific types of AML [4].

The human DNA methyltransferase gene (DNMT1,DNMT3A and DNMT3B) encode enzymes which catalyze the addition of a methyl group to the fifth position of cytosine,generating 5 methylcytosine, by this mechanism, the DNA methyltransferase mediate the downregulation of target gene via the methylation of upstream CPG islands. Gene mutation in DNA methyltransferase which alter enzyme function have now been described in acute leukemia, Yamashita et al. reported [5] 4.1% somatic mutation of DNA methyltransferase (DNMT3A) at amino-acid Arg882 in leukemia, Later Ley [6] et al. found 22.1% DNMT3A mutations in adult normal karyotype AML genome and Chen SJ also discovered gene mutations in DNMT3A in patients with M5 (20.5%) and with 13.6% of M4 subtype [7]. those results above indicated that DNMT3A mutations were independently and significantly associated with decreased survival in adult AML. in order to analyze the influence of DNMT3A mutation on the prognosis and survival of AML. In this study, RNA from bone marrow cells of untreated AML patients from the Chinese patients were examined by using PCR and sequence analysis, simultaneously chromosome examination and immunophenotype were also performed.

## Method

### Patients and samples

There were 101 patients aged 16–76 years with AML in this study, those were newly diagnosed AML which were classified according to French-American-British(FAB) classification system, including M0(n = 2),M1(n = 4), M2(n = 41), M3(n = 25), M4(n = 6),M5(n = 21), M6(n = 2). All of them were diagnosed and treated in the Zhongshan Hospital of Xiamen University from July 2008 to Dec 2010. Bone marrow(BM) aspirates or peripheral blood(PB WBC > 50 × 10^9^) sample were collected at the first visit and in accordance with guidelines from the ethics committee of the hospital.

### Cytogenetic analysis

Bone marrow cells were harvested directly before chemotherapy according to the study [8] , the metaphase chromosomes were banded by the G-banding techniques and karyotyped according to the International System for Human Cytogenetic Nomenclature.

### Immunophenotyping

A panel of monoclonal antibodies to myeloid-associated antigens including CD13, CD14, CD33, as well as lymphoid-associated antigens including CD2, CD5, CD7, CD19, CD20, and lineage nonspecific antigens HLA-DR, CD34 were used to characterize the phenotypes of the leukemia cell with Epics XL4 flow cytometer (Beckman Coulter, America).

### RNA preparation

Total RNA was isolated using RNAprep pure Blood Kit (Tiangen Biotech, China), according to the manufacturer’s instructions,followed by treatment with RNase-free DNaseI to remove contaminating genomic DNA. The RNA was dissolved in RNase-free H2O, checked for purify and concentration with ND-1000 UV–vis Spectrophotometer (Nanodrop Technologies, USA) and stored at -80°C until to be used.

### Reverse transcription PCR(RT-PCR)

Reverse transcription was performed with T3 Thermocycle (Biometra, Germany), total RNA (2 μg) was mixed with 10 mmol dNTPs and 25 pmol random primers (TOYOBO, Japan), incubated at 65°C for 5 minutes, chilled immediately on ice, followed by addition of 20 U RNase inhibitor, 1 RT buffer, 100 U MMLV reverse transcriptase (Sangon, China) and RNase-free water to make up a volume of 20 μl. RT reaction was incubated at: 37°C 10 minutes; 42°C 60 minutes; 70°C 10 min. Resultant complementary DNA (cDNA) was diluted with 180 μl ddH2O (10 ng/ μl RNA equivalent) and was stored at -20°C.

### Mutation analysis

The DNMT3A was amplified by 2 pairs of primers and sequenced by the same ones. The sequences of these PCR and sequencing primers are F1: 5’-GCCACCAGAAGAAGAGAAGAATCC-3’,R1: 5’-CTCTTCTGGGTGCTGATACTTCT-3’,F4: 5’-CTCGCCTCCAAAGACCACG-3’,R3: 5’-TTCTCCGCTGTGCTCTTCC-3’.

The reference sequence was NM_022552.3 for mRNA. In every 25 μL of PCR reaction, there were 1 SSP buffer (67 mM Tris–HCl, 16 mM (NH4)2SO4, 0.01% Tween 20, pH 8.8), 0.2 mM dNTPs, 2.5 mM MgCl2, 0.4 uM each tagged primer, 0.5 U TaqHS DNA polymerase (Takara, Japan), and 5 μl of cDNA. The amplification was performed in a Thermal Cycler PCR instrument (Bioer, China) under the following conditions: 95°C 3 minutes; 10 cycles of touchdown PCR, 95°C 20 seconds, 65°C -60°C (decreased by 0.5°C per cycle) 20 seconds, 72°C 90 seconds;30 cycles of 95°C 20 seconds, 60°C 20 seconds, 72°C 90 seconds and finally,72°C 7 min.The PCR products were verified by electrophoresis and sequencing. Clone the DNA fragments with overlap peak into pMD18-T vector (Takara, Japan) and sequencing.When the mutations were not obvious because of location near the primers,sequencing from the other direction was done to solve this issue.

### Statistical analysis

The discrete variables of patients with and without gene mutation were compared using the χ2 tests, but if the theoretical frequency was smaller than 5,correction for continuity was used All statistical analyses were performed with SPSS 16.0 software(SPSS Inc).

## Results

### DNMT3A mutations

**Figure 1:**
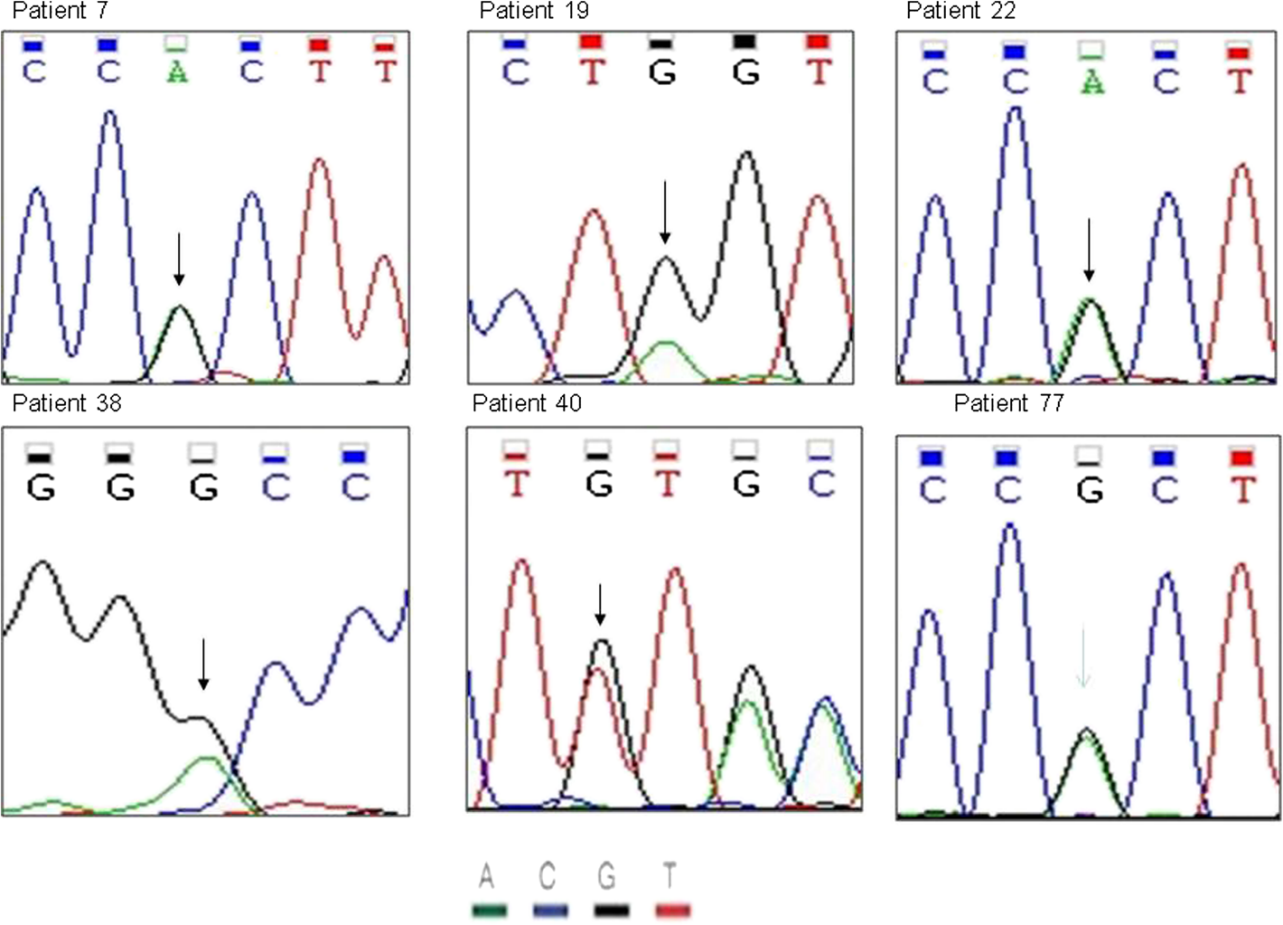
Sequencing results for patient 7(2645 G > A), patient 19(914 G > A), patient 22 (2645 G > A), patient 38(2906 G > A), patient40 (30 bp del 2502-2531 bp) and patient 47(2501 C > T), Patient 40 harbors a 30 bp deletion and shows a overlap peak.

**Figure 2:**
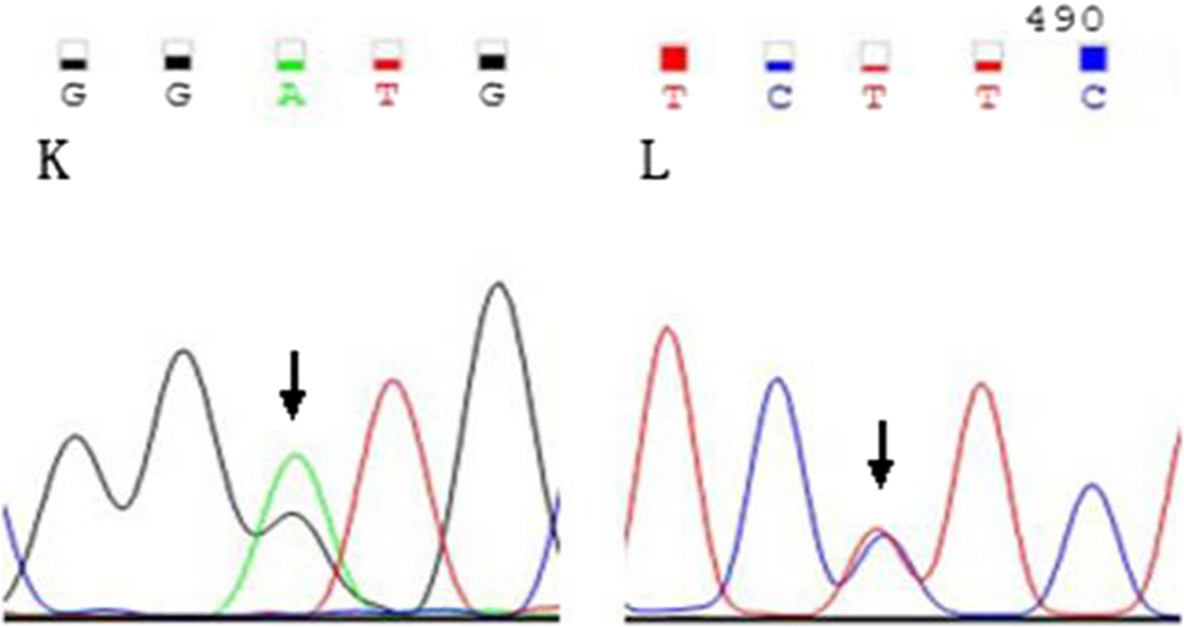
Sequencing results for patient 89, the double point DNMT3A mutation was K: 1906 G > A,L:2191 T > C.

In my study, 101 samples from new diagnostic AML were detected by PCR amplification of entire coding region of DNMT3A, the detection rate of DNMT3A mutations in AML patients was 13.9%(14/101), including 2 patients of M1, 2 patients of M2,1 patient of M3 and 9 patients of M5(42.9%). (Table1) .the type of mutations was 11 missense, 2 nonsense and 1 deletion mutations.respectively. single nucleotide variations included 2645 G > A(Arg882His), 914 G > A(nonsense), 2096 G > A(Gly699Asp), 2501C > T(Thr834IIe), 55C > T(Arg19Trp), 2202C > A(Phe734Leu) and 2644 C > T(Arg882Cys).(,Fig-1) The most common mutations are predicted to affect amino- acid Arg882 (in 6 patients) and Thr834(in 2 patients).We also detected a 30 bp deletion according to sequencing (Figure 1, patient 40). Double point mutation was also detected in 2 patient (Figure 2).DNMT3A mutation (2645 G > A) was found in BM sample of the old patient with M5, this is the same with that of Ewalt reported (Figure 1 patient 77) [9].

**Table 1:** Clinical features of the Patients with DMNT3A mutations

**No**	**Age**	**sex**	**FAB**	**Cytogenetics**	**WBC X10^9/L**	**CD33+/CD34+**	**DNMT3A**	**Chemo-response**	**result**
7	65	F	M1	normal	36.6	6%/92%	2645 G > A	CR	Dead(12 M)
19	72	M	M2	normal	57.2	99%/32%	914 G > A	No	Dead(6 M)
22	55	M	M5	normal	112.9	99%/16%	2645 G > A	CR	Alive(SCT)
38	58	F	M5	t(6;9)	12.1	56%/55%	2096 G > A	CR	Dead(10 M)
40	56	F	M2	inv(9)	15	20%/82%	30 bp del 2502-2531	CR	Dead(SCT)
47	31	F	M5	del(13)	1.5	95%/27%	2501 C > T	N0	Dead(3 M)
48	20	F	M5	normal	30.4	4.5%/71%	2501 C > T	N0	Dead(3 M)
53	53	F	M3	t(4;17)	7.9	82%/1.6%	55 C > T	CR	Alive
56	54	F	M5	normal	2.19	95%/28%	2202 C > A	CR	Relapse(6 M)
68	52	M	M5	normal	28.8	97%/42%	2644 C > T	No	Dead(6 M)
77	76	M	M5	normal	67	No	2645 G > A Arg882His	CR	Dead(13 M)
89	76	M	M1	normal	17.3	CD13 CD34	1906 G > A 2191 T > C	NR	Dead(3 M)
94	61	F	M5	normal	132	CD7CD13CD33	2141C > G	NO	Dead(1 M)
100	46	M	M5	normal	4.5	CD13CD34	1792C > T 2375 G > A	NR	Dead(1 M)

*CR* Complete remission, No no chemotherapy, *NR* not remission, *M* month.

### Clinical features of DMNT3A mutation positive patients

Of the mutated patients, AML-M5 subtype had higher DNMT3A mutation rate than those with other subtype. Cytogenetic data showed that 10 cases with DNMT3A mutation was normal karyotypes AML patients (71.45%,p > 0.05). No DNMT3A mutations were found in AML samples with cytogenetic findings associated with a favorable (except 1 patient with M3). In all of positive patients, 8 patients were female (57.1%, p > 0.05), 11 patients were older than 50 years (78.6%, p < 0.05). we also discovered that DNMT3A mutations were associated with high expression of CD33 or CD34, After having been treated by induction chemotherapy, all patients achieved complete hematological remission(76.5%), but 4 patients refused to receive chemotherapy, after 12 monthes follow up, only 2 patients were alive including 1 M3 patient.

## Discussion

Epigenetic mechanisms play a crucial role in regulation of gene expression and DNA methylation catalyzed by specific DNA methyltransferase and DNMTs is an important epigenetic control of gene expression and the maintenance of genome integrity. Many DNMTs appear to be present in human and have a different role in establishing and maintaining the DNA methylation pattern including DNMT1, DNMT2, DNMT3A, DNMT3B and DNMT3L [10,11]. DNMT1 is the primary enzyme responsible for copping methylation patterns after DNA replication while DNMT2 does not methylate DNA but instead methylates small RNA,. DNMT3A and DNMT3B are paralogous enzymes responsible for de novo DNA cytosine methylation. DNMT3L plays a key role in allowing DNA methylation during the maturation of germ [12]. Mouse studies showed that DNMT is required for hematopoietic stem cell self renewal, regulation of proliferation and normal myeloid and lymphoid differentiation. By contrast, deletion of DNMT3A and DNMT3B in mouse hematopoietic stem cells impaired stem cell self renewal, the frequent DNMT3A mutations may be an parts of pathogenesis in acute myeloid leukemia [13].

In this study, we report the frequency of DNMT3A mutations in patients with de novo AML and their Clinical features compared to the patients without DMNT3A mutations. We sequenced all coding exon of DNMT3A using DNA from leukemia cells and identified 14 heterozygous mutations (13.9%). Amino acid R882, located in the methyltransferase domain of DNMT3A, was the most common mutation site. Our results showed that the rate of DNMT3A gene mutations in Chinese AML patient is more common and AML-M5 was more higher than other type(9/21) the alterations of Arg882 are also the most frequent mutation in Chinese AML patients. We also found one AML-M3 patient who carried t(4; 17) chromosomal abnormality carry DNMT3A gene mutation and this is not reported by other papers [6,7].

Our results indicated that most of patients(11/14) with DNMT3A mutation died of leukemia after 6 months treatment, two patient were alive due to allogeneic stem cell transplant and AML-M3 type. The last one was one relapse. Although it is unclear if DNMT3A mutation can affect survival of AML patient, this fact was accord with concept that DNMT3A mutation could be a dependent factor on AML patients. In concurrence with other reporters, there was no significant association between DNMT3A mutations and sex, high WBC or increased blast percentage in the bone marrow at diagnosis. But the high expression of CD33 or CD34 on AML cells was very common., DNMT3A mutation were found in patients above 50 years of age, this is agreement with previous studies. Although DNMT3A gene mutation was almost never found in AML-M3 subtype, this patient carried t(4; 17) chromosomal abnormality and PML-RARα fusion gene, it may be associated with a poor prognosis for no-classical acute promyelocytic leukemia.

To conclude, it seems that DNMT3A mutations are consistently present in approximately 10% of AML cases, the role of DNMT3A mutations on leukemogenesis was still unclear. Future studies will help to illuminate the pathogenesis of AML, we need to develop a method of quick determination of the whole gene mutations and evaluate the influence of DNMT3A mutation on treatment response and survival in a larger sample.

## Competing interest

The authors declare no financial or commercial conflict of interest.

## Authors' contributions

Dr Quanyi Lu organized the research plan, Dr Yamei Chen analyzed data, performed experiments and drafted the paper. and Dr zhipeng Li and Prof Hang Wang coordinated the study, participated in its design and contributed to writing. All authors read and approved the final manuscript.
